# Intellectual disability nurses’ challenges in medication management in primary health care: A qualitative study

**DOI:** 10.1177/17446295231189368

**Published:** 2023-07-12

**Authors:** Elfrid Måløy, Maria Therese Aasen- Stensvold, Solfrid Vatne, Signe Gunn Julnes

**Affiliations:** Department for Health and Social Care, 5562 Molde University College, Kristiansund, Norway; Intellectual Disability Nurse, Kristiansund Municipality, Kristiansund, Norway; Department for Health and Social Care, 5562 Molde University College, Molde, Norway; Department for Health and Social Care, 5562 Molde University College, Molde, Norway

**Keywords:** intellectual disability nurse, medication management, intellectual disabilities, residential care homes, primary health care, qualitative method

## Abstract

This study examines how intellectual disability nurses employed in residential living services for persons with intellectual disabilities, in Norway, deal with medication management for these individuals. Using a qualitative study, a total of 18 intellectual disability nurses were interviewed as part of four focus groups. The results demonstrate six main challenges: First, Being alone with the responsibility of medication management - a challenge; Second, The need for further competence development; Third, Teaching and supervising unskilled colleagues in safe medication management; Fourth, Interpreting residents with little or only nonverbal communication; Fifth, The need to act as advocates when residents require hospitalization; Sixth, Deficient systems for medication management on several levels. The findings point to several major flaws in the system of medication management, which necessitates the need for highly qualified intellectual disability nurses. Managers must ensure that there is a secure system to mitigate errors and promote patient safety.

## Introduction

### Intellectual disability nurse

Intellectual disability nurses, “Vernepleier” in Norwegian, are caregivers to persons with an intellectual disability. Intellectual disability nurses have a specific education consisting of a three-year full-time degree of Bachelor of Science Course, 180 European Credit Transfer and Accumulation System (ECTs), (Norwegian Ministry of Education and Research, 2019).

Intellectual disability nurses play an important role in working with people with intellectual disability, usually in their residential living services-part of the primary health care services. The Intellectual disability nurses have a special professional competence in holistic care with person centered approaches for the needs of people with intellectual disability (Brown et al., 2016; Grong, 2019). The intellectual disability nurse’s profession has combined health and social work, with an interdisciplinary approach based on health sciences, social sciences, law, pedagogy, and psychology (Grong, 2019). Medication management training is included in the education. Among several professional tasks, the intellectual disability nurses also are authorized health personnel with the responsibility for the proper handling of medication required for people with intellectual disability (Ellingsen, 2019; Norwegian Directorate of Health, 2015). Safe medication management involves effective measures that safeguard the quality of patient safety (Ministry of Health and Care Services, 2012).

### Persons with intellectual disabilities

The World Health Organization (WHO) points out that between 1 and 3 percent of the population has an intellectual disability, which in Norway is between 47,000 (0.95 %) and 61,500 (1.23 %) people (NAKU, 2019; Norwegian Directorate of Health, 2019). In 2016, approximately 19,000 people with intellectual disability received health services in Norway (Norwegian Directorate of Health, 2019; NAKU, 2019). Persons with intellectual disability may frequently be transferred through a range of healthcare sectors, such as hospitals, outpatient clinics, and municipal home-based healthcare services. However, most people with intellectual disability live in residential homes with 24-hour care (Erickson, 2020; Halmetoja et al., 2023; Norwegian Directorate of Health, 2019; Sheerin et al., 2021). Intellectual disability is diagnosed based on three criteria: (1) IQ lower than 70, (2) difficulties with language, motor skills and coping with daily life, and (3) the condition must be diagnosed before the age of 18 (Norwegian Directorate of Health, 2019). People with Intellectual disability may have problems expressing concerns about their health. Their ailments include congenital health issues and are more frequently diagnosed with comorbidities compared to the general population. Furthermore, people with intellectual disability have an earlier mortality rate than people without intellectual disability (Langballe et al., 2017; Norwegian Directorate of Health, 2019).

A study by Hove et al. (2019) showed that persons with intellectual disabilities use more medication than other persons, and that many of them may have difficulties in expressing the need for and effect of the medication they use. The study by Scheifes et al. (2016) showed that psychotropic medications are prescribed to approximately 30%–40% of adults with intellectual disability who display challenging behavior. This medication may have one or several side effects that negatively impact the residents’ quality of life.

### Medication management for persons with intellectual disability

The term “Medication Management” is defined in Norwegian regulations as: “Any medication-related task performed any time during the prescribing or requisitioning of the medication until its eventual distribution to the person requiring the medication” (Norwegian Directorate of Health, 2015). This means that everyone who is to dispense medication, must have training in medication management as a prerequisite. This includes, but it is not limited to, observation during administration. To delegate this responsibility, a quality-assurance system is required that promotes patient safety (Norwegian Directorate of Health, 2015).

It is the primary health service’s responsibility to ensure all medication management tasks involving people with intellectual disability carried out by private companies or service providers follow strict protocols. There are guidelines that must be followed by employees while performing medication management tasks. The management of the residential care homes must ensure that the employees who handle medication have the necessary training and professional competence. The list of medications is prescribed and updated by the resident’s general practitioner (GP). When transferring between the GP and the hospital, the handover must include the complete medication list (Norwegian Directorate of Health, 2015, 2020). The authorities require sufficient competence in medication management among health personnel (Norwegian Directorate of Health, 2015), and that health institutions that provide health services offer employees training and further education in medication handling. In addition, health personnel have an individual responsibility for acquiring new knowledge in their profession and keeping themselves updated (Norwegian Directorate of Health, 2015).

Studies show that there is a higher incidence of excessive use of medication among elderly multimorbid and frail people and people with challenging behavior with intellectual disabilities (Erickson et al., 2022; O'Connell et al., 2020; O’Dwyer et al., 2018). Frequent errors have been reported when administering medication to people with intellectual disabilities, which might have serious consequences. Inadequate guidelines and frameworks for medication management have been identified within a complex area of practice (Joos et al., 2015; Sheerin et al., 2021). A study by Erickson and Yang (2019), where the intention was to investigate challenges in medication management reported by caregivers to adults with intellectual disability, showed that the caregivers experienced stress and anxiety when administering medication to the intellectual disabilities. There was a lack of training on the use of medication among the employees, furthering the need for closer collaboration with pharmacists who can offer advice on the complexity of medicine regimens, thus avoiding polypharmacy.

Another study by Auberry et al. (2019) emphasized that medication management can be better with training among caregivers through use of simulation, debriefing, and reflection. The researchers believed there is a need for further studies that consider the unique problems emerging in the medication management of people with intellectual disability (Joos et al., 2015; Sheerin et al., 2021). Several studies ask for a focus on how to understand why errors occur during the medication management process and how these can be addressed (Joos et al., 2015; Peklar et al., 2017). Furthermore, research is needed to gain knowledge about targeted quality improvements. It is important to understand what problems intellectual disability nurses face throughout the medication management process and how these can be addressed (Erickson et al., 2016; Joos et al., 2014).

For several decades, intellectual disability nurses in Norway have had an important role in medication management for people with intellectual disability in residential care homes. However, a search of several databases shows a lack of research on how this is actually managed. It is timely to explore this important area of the intellectual disability nurse’s role, and the challenges they may have managing medication for people with intellectual disability.

The research question is: What challenges do intellectual disability nurses experience in medication management of persons with intellectual disability?

## Method

### Focus group interviews

According to Krueger and Casey (2015), focus group interviews are a suitable method for collecting data. It reflects the diversity of experiences among several participants on a specific topic. Unlike in-depth interviews, the data is generated from a group through a dynamic interaction process. Consequently, we designed semi-structured interviews in line with Kvale et al. (2009), in order to identify the intellectual disability nurses’ experiences with medication management for residents with intellectual disability living in residential care homes.

### Recruitment and sample

The criteria for inclusion required experiences with medication management for intellectual disability nurses who work in residential care homes. Altogether, the residential living services had approximately 130 intellectual disability nurses meeting these criteria from four municipalities with 75,000 inhabitants. Managers working at four different care homes, from two different urban municipalities and two different cities, helped us in the recruitment of intellectual disability nurses. The managers delivered consent forms and letters containing information about the purpose of the study and the consent form to intellectual disability nurses. In total, eighteen intellectual disability nurses -fifteen women and three men-volunteered to participate. Five of the 18 participants had 15 credits in medication management at a master's level. They were aged between 25-60 years and their work experiences ranged from one to more than 25 years. The participants were divided into four homogeneous focus groups from different urban municipalities to bring out variations in the experiences (Krueger and Casey, 2015).

### Implementation of the interviews

The first author moderated each focus interview, while the fourth author, the co-moderator, followed the course of the discussions and took notes. To ensure confidentiality, all participants were given pseudonyms to de-identify the informants during the interview. The quotes are anonymised in the findings. We use the term intellectual disability nurse in presentation of findings (Krueger and Casey, 2015). Each interview lasted one hour and was audio recorded. The interviews were transcribed verbatim after each session. Personal details of the interviewees were removed from the transcript and checked against the moderator's and co-moderator's notes to verify the identity of the speakers on the tape.

### Data analysis

We analyzed the transcribed interviews according to qualitative content analysis, inspired by Graneheim and Lundman (2004), which consists of multiple levels of abstraction of two different forms, the manifest and the latent. The manifest describes the visible, literal content of the text and the data material, with a focus on what the text says. The latent part of the analysis, presented as a transition from meaning units, condensed meaning units to codes, subthemes, and themes, is considered the researcher's interpretation of the original data material (Graneheim and Lundman, 2004). The authors conducted the analysis with the following five steps: 1) each interview transcript was read several times to identify preliminary themes; 2) the text was then divided into units of meaning and the content of the direct quotes was extracted and condensed using the participants' own language; 3) the condensed units of meaning were marked with codes to organize the data; 4) the codes were compared, similarities and differences were identified, and a structure of themes and subthemes was created; and 5) a comprehensive understanding of what we had summarized and reflected on from the findings was described as an overall latent theme (Table 1).

**Table 1.** Example of the analytical process.

Meaning units	Condensed meaning units	Codes	Subtheme	Theme
Also, on the weekends there is only one intellectual disability nurse on shift, and if wrong medication is given or one has forgotten to give warfarin (anticoagulant), I must call and ask the physician in the emergency room, who does not know the resident. There is a lot of responsibility on your shoulders.	At times when only one intellectual disability nurse is working, and if wrong medication is given or forgotten, they seek advice from the emergency room, who does not know the medical history of the resident.	Alone with the medication handling.	Consulting physician who does not know the resident.	Being alone with the responsibility of medication management - a challenge
		Medication errors occur.		
		Consulting physician.		
		Emergency room does not know the case history of the residents.		

## Results

The results revealed that intellectual disability nurses experienced a great deal of responsibility for the medication management process, with complex challenges at different areas and levels. The results are presented in the following six themes and two subthemes.

### Being alone with the responsibility of medication management - a challenge

Intellectual disability nurses expressed concerns that they were left alone with the responsibility for handling medication. Some revealed they performed medication tasks very rarely, but when called upon, they were expected by the managers to do so competently and adhere to stringent health and safety protocols. Another challenge was having very few colleagues with the same or similar qualifications to perform quality checks with during administering of medication. It was also revealed that the staffing levels of intellectual disability nurses was too low to meet the demand in terms of administration and sheer volume of the medication management needed.

Furthermore, colleagues were often unskilled care workers without an education in healthcare, and with minimal experience in medicine. One intellectual disability nurse expressed:

I feel that I have a huge responsibility to make sure that the residents receive the right medicine at the right time and administered correctly. It is of utmost importance that our unskilled colleagues are trained in this because they are here more often than we are.

As a new intellectual disability nurse employee or graduate, this situation is particularly challenging because they are not yet familiar with the effects of certain medications on residents. Another intellectual disability nurse expressed: “I find it especially concerning when I don’t have enough knowledge of all the residents at the same time as I am responsible for their medication management.”

Intellectual disability nurses revealed that they do not have a physician employed at the residential care home. They expressed that when a physician was needed, often the consulting was conducted via e-mail. One intellectual disability nurse said:

It was challenging, especially in the weekends, to be alone. Physicians who know the residents were not working and it was not a good feeling to care for someone with a high risk of clinical deterioration.

Doing weekends, or when physicians were not working, they resorted to calling emergency services for medical opinions. One of the intellectual disability nurses said:

Also, on the weekends there is only one intellectual disability nurse on shift, and if wrong medication is given or one has forgotten to give warfarin (anticoagulant), I must call and ask the physician in the emergency room, who do not know the resident There is a lot of responsibility on your shoulders.

Intellectual disability nurses experienced a great deal of responsibility and described this situation as psychosomatic stress. One of the intellectual disability nurses uttered: “Sometimes on weekends you go to work feeling tense. It’s awful.” They said that they had to ensure that the residents received the right medicine and reported on the medicines that can provide interaction, thus ensuring the quality of care of the residents.

### The need for further competence development

Even though the intellectual disability nurses were educated in medication management, on occasion they felt insecure about their abilities. For example, they lacked practice of intramuscular medications. They revealed that the quality of safety procedures in medication management was insufficient, and they wanted further training. One intellectual disability nurse said:

It is clear that within our field of work there is a lack of knowledge in medication management. We are struggling with that. The policy here is, if you have a Bachelor of Science in Intellectual Disability Nursing, you should be able to handle it, but I lack confidence, and if something goes wrong, it is my responsibility …

They expressed that they had to take personal responsibility for their own professional development within medication management. Several intellectual disability nurses revealed that they did not have sufficient pharmacological knowledge stating the need for a refresher course in medication management, which was not offered by management. As one intellectual disability nurse told:

We had training in medicine handling in the laboratory during our education, and it was worth its weight in gold. If you get a lot of medicine-related tasks, you will gain experience, if not then refresher courses should be offered.

The training was poorly organized with lack of planning in medication handling. The workplace offered training via online courses but was often unsatisfactory. However, some stated that they benefited from these courses which allowed them to stay up-to-date with medication management procedures. Some suggested an internship with experienced nurses at a nursing home. A previous nursing home employee expressed that she learned a lot about medication management from the nurses: “I previously worked as an intellectual disability nurse in a somatic nursing home for a year and I have never learned so much as I did during that time.” Some expressed that they had higher education in medication management and become more confident:

I feel more confident after I sought further education in medication management with a focus on improvement work in my own workplace. I learned a lot there, and I developed a critical eye for the role.

### Teaching and supervising unskilled colleagues in safe medication management

When intellectual disability nurses were not on duty, unskilled health care workers, social workers, teachers, all without professional training in medication management, were delegated the responsibility to distribute medicines to residents. Because of a lack of standardized training and dictated routines, intellectual disability nurses expressed that it was important for them to teach colleagues with insufficient knowledge. They revealed that they felt a great responsibility to ensure that there were no errors or deviations with medication that could harm the residents. The intellectual disability nurses recognized the importance of teaching colleagues about medication management and informing them about specific resident’s medication use. A participant put it this way:

We often get questions from unskilled health care colleagues about which medication the resident should have, and why they should have this medication. Our responsibility is to answer in a clear and concise manner-explaining their individual treatment plans and why it is so important that the residents take the medicine at the right times.

The intellectual disability nurses stated that an important part of the training of an unskilled employee is easy-to-understand written procedures. A participant elaborated: “Out of necessity, I have created a training procedure and booklet for our workplace. I have listed different medications and their side effects.” Intellectual disability nurses with further education in medication handling, expressed that this competence had made them aware of the lack of safety measures around the handling of medication in the workplace.

### Interpreting residents with little or only nonverbal communication

Intellectual disability nurses highlighted that through their education they had acquired the knowledge and skills to interpret and understand people with different types of intellectual disabilities. As part of their practice, they have to interpret the behavior of the residents who find it difficult to communicate, especially after taking their medication.

#### The importance to observe and assess the effects of the medication

Intellectual disability nurses’ level of competency helped them to recognize the effects and side effects of medication on residents. An intellectual disability nurse said:

It is very important to observe and get to know residents well. Some residents cannot communicate verbally and sometimes it seems that the medication has no effect on their agitated state. While other times the effect is clearly evident; the resident enters a sedated state after a period of time.

It was expressed that identifying, and as a result, understanding the degree and location of the persons’ pain required knowledge and familiarity with the resident's condition:

It is important to recognize if the residents are in pain, the cause of pain, and why they react in a certain way; for example, facial expressions, type of pain and severity through their body language.

When a resident showed physical aggression and uncontrolled behavior, a challenge was deciding whether to administer sedatives, and the dosage - a physician prescribed medication, but it was the intellectual disability nurse who was responsible for assessing whether the resident needed medication when a specific behavior occurred. An intellectual disability nurse explained:

[She] attacked us, spat, hit, scratched, and kicked. It took two members of the staff to restrain her. Our role does not involve medicating persons, but when you have held down a person for six hours, you must consider using sedatives like Diazepam to aid in calming down the residents. Often it does not work, it has no effect. If this is the case then you must consider if it is appropriate to increase the dose, and by how much. This situation was very difficult.

The intellectual disability nurses also expressed that it could be difficult to assess whether the change was a worsening of the condition, or a side effect of medication or changes in relation to the diagnoses - which could happen in some cases.

### The need to act as advocates when residents require hospitalization

The intellectual disability nurses provided several examples where they had to be an advocate for an intellectual disability person when there was a need for health services from other health professionals outside their home. There were instances of residents on warfarin (anticoagulant), who were also given Ibuprofen. One participant said that “It has happened often.”

The intellectual disability nurses expressed that even though these medicines were contraindicated due to an increased risk of bleeding, the medicine was given in the emergency room. To avoid such mistakes, they therefore had to be immediately consulted on what was to be given in the emergency room.

The intellectual disability nurses stated that when the residents were hospitalized, they must be present during the entire hospital stay as advocates and as the source of information pertaining to their resident’s medical treatments: “Although we will only be advocate for the resident during the hospitalization, they left the responsibility with the medication to us intellectual disability nurses who knew the resident.”

### Deficient systems for medication management on several levels

The intellectual disability nurses expressed that the system at the workplace was not well designed enough to ensure patient safety for medication handling. A participant expressed:

The medication handling area must be a suitable, purposeful section. In our workplace, there is a worktable for the task, but it’s surrounded by a busy hallway, office and a break/common room. There is a lot of noise and disturbance from people passing by, meaning we cannot adequately concentrate on medication handling.

A major dilemma for the intellectual disability nurses involved experiencing cross-pressure between competing tasks that made it difficult to concentrate on the handling of medicines. They wanted time allocated for sorting medication. Several expressed that there was a lack of understanding among the other employees during medication handling:

No, we do not have a dedicated amount of time for focusing on medication management. Often you are interrupted, causing one to make mistakes. We are forced to prioritize this task ourselves and can only try and find the time, rather than have a set period to perform the task. I have even had to race to perform another task and accidentally left the medicine cabinet open. Occurrences like this could be avoided with a clear and organized schedule.

The general consensus was that there were inadequate routines and schedules for controlling medication, with some recalling that no double checking was carried out before medication was given to the residents. One participant said:

We have a locker with locked medicines for everyone and then it's a bit occasional who of us have the control. We do not have a special fixed plan for that. So, it can quickly get a little messy.

#### Various challenges leading to medication errors and deviations

The intellectual disability nurses had experienced that various errors - incident reports were inadequate, were not registered or were not followed up in the workplace. During the medication usage processes, on occasion, medications were not given on time, wrong medicines were given, or they were not given at all. Several intellectual disability nurses from different residential care homes emphasized that the residents repeatedly did not receive their regular medication, because they were forgotten. These incidents were not registered as deviations and doubt was cast whether the medication was given or not. An intellectual disability nurse described it as follows:

No medication was given for two days in a row, or when medication was given, the doses were incorrect. In the case of Warfarin, missing doses is a risky thing, as it’s a blood-thinner.

An incident could occur when several people are responsible for the process of medication management (dispensing stage), from the medicines being taken out of the medicine cabinet, to then being given to a colleague responsible for giving it to the user. Guidelines for who should be responsible for signing the medication form were unclear. Participants expressed that they wrote incident reports when errors and non-conformities occurred in handling of medicines. However, the challenge was that it was not followed up by the management, and thus there were no improvements in the handling of medications.

## Discussion

### Lack of safety in medication management

The findings in the present study show unsafe medication handling practices for the residents in several areas. Our findings show that the intellectual disability nurses experienced being alone with the main responsibility for medication management in residential care homes. They are concerned about the lack of quality safety measures both before and during the distribution of medication. This is supported by the study of Erickson et al. (2016), showing a lack of accuracy in medication handling, i.e. medicine lists. The guidelines stated in the Medicines Handling Regulations (Norwegian Directorate of Health, 2015) describes that checks should be carried out by two people who have competence in the preparation of medicines. Double checking is an important measure to reduce the risk of errors (Storli et al., 2016). Our findings show that this is not possible when left alone on duty. According to Ellingsen et al. (2020) only 10.7% are educated as an intellectual disability nurse who works with people with intellectual disability. Fellesorganisasjonen (2020) has revealed a serious shortage of intellectual disability nurses. There are today approximately 12,500 intellectual disability nurses in Norway, and there is a shortage of over 20,000. This means that on several shifts throughout the week, there is a shortage of intellectual disability nurse workers. Residents may have complex conditions and are prescribed more medication than other patient groups. This requires that intellectual disability nurses are present and have updated knowledge within the subject area (Hove et al., 2019).

Our study shows that the intellectual disability nurses take their responsibilities seriously and follow up to check that the residents receive the right medicine. When errors are discovered, they contact the physician for confirmation of the correct medicine list. Productive interaction between physician, an intellectual disability person and a caregiver leads to appropriate treatment decisions, including the writing of prescription. An unproductive relationship or a complete lack of communication between physician, an intellectual disability person and their caregiver allows for incomplete or inaccurate assessment of medical problems and situations like this to occur (Mastebroek et al., 2014).

Another finding was that intellectual disability nurses still felt responsible for residents even when they were not on duty, and especially if unskilled colleagues were in charge of distributing medication. Even though their colleagues had taken medication courses, the intellectual disability nurses felt that they were not qualified enough to have this responsibility. This is in line with a study by Ellingsen et al. (2020), who found that in residential care homes there was low competence and extensive use of part-time workers. They concluded that the requirement for professional soundness presupposes that the proportion of intellectual disability nurses must be increased significantly. According to the Medical Management Regulations (Norwegian Directorate of Health, 2015), the management of the residential care homes must ensure that employees who do not have appropriate competence are only given tasks, such as distributing medicines. However, it is not enough to just distribute the medicine according to the regulations, but professional observations of the resident's reactions are required. This means that everyone who is to dispense medication, as a minimum, must have training in the relevant medication handling, including observation during administration (Norwegian Directorate of Health, 2015). Van Den Bemt et al. (2007), shows that many errors occur at the administration stage of the medication distribution process among employees who are working with persons with intellectual disability.

### Management must facilitate skill-development

The intellectual disability nurses in our study expressed that there is a need for professional development and further education within medication management among intellectual disability nurses, especially in places where handling of medication is rare. Several studies support the refreshing of knowledge on medication management on a regular basis, both theoretically and practically, to ensure patient safety (Erickson, 2020; Storli et al., 2016; Wannebo and Sagmo, 2013). There were only offers of e-learning courses. These produced varying degrees of acceptable outcomes. There is a strong desire that management regularly facilitate up-to-date courses. In a study by Auberry et al. (2019), they found that training with the use of simulation, debriefing and reflection with medical management resulted in good learning outcomes and an increase of knowledge and understanding. A quantitative study of comparable professions by Wannebo and Sagmo (2013), revealed that nurses in nursing homes had a great need for training in pharmacology. Nearly 100% of 262 nurses agreed that courses should be given to employees responsible for medication handling, and 97% thought that it should be a mandatory course. According to Zaal et al. (2016) and Peklar et al. (2017), intellectual disability nurses who are responsible for medication management must have good professional knowledge and observational skills regarding diseases, especially in elderly intellectual disability persons, since they have a higher morbidity and require a higher number of medications compared to other demographics. The Medication Management Regulations emphasize that the responsibility with medicines entails having a comprehensive knowledge of the field to ensure patient safety (Norwegian Directorate of Health, 2015, 2020). To facilitate training, it is important to understand what problems intellectual disability nurses encounter throughout the medication treatment plan process and how these can be solved (Erickson et al., 2016; Joos et al., 2014).

### Intellectual disability nurses’ personal accountability when training colleagues in medication management

The findings of our study showed that since intellectual disability nurses were not present around the clock, they felt obligated to train unskilled colleagues in medication management to ensure safety of the residents. The intellectual disability nurse has supervision and teaching responsibility in their profession. According to The Norwegian Board of Health Supervision (2017), unskilled employees and temporary employees given tasks to distribute medicines may cause serious errors. The Norwegian Social Educators and Social Workers' Association (Fellesorganisasjonen, 2020), points out that there are too few intellectual disability nurses employed in residential care homes. Intellectual disability nurses who had further education in medication management expressed that the education led them to take responsibility for improving procedures in medication management. According to a study by Auberry et al. (2019), it is important that employees receive training in understanding medical processes, causal relationships, and consequences of medication. According to Ellingsen et al. (2020), we take it for granted that health personnel are authorized and have medical education, but the reality is there are too few with intellectual disability nurse education. A Norwegian Official Report (NOU, 2016) describes the need to realize basic rights to ensure professional health care services for people with intellectual disability. Over the years, the committee has proposed increasing intellectual disability nurses for promoting greater professional security and responsibility for medication management.

### The importance of observing the residents and knowing them well

One of our research findings has shown the importance of the role of education in intellectual disability nursing in providing skill sets for communicating and understanding people with different types of intellectual disabilities. Furthermore, the finding has demonstrated that adequate training can help intellectual disability nurses correctly identify and assess the effects and side-effects of medication. The intellectual disability nurses also emphasized the importance of observing the behavior of the residents and knowing their medical history. A review by Martin et al. (2010) emphasized intellectual disability nurses’ expertise in interpreting intellectual disability persons’ non-verbal communication to promote understanding. Poor communication is perceived as a barrier to effective health services for both health professionals and people with intellectual disability.

In our study, intellectual disability nurses reported struggling with reliable pain assessment for pain relief towards people with intellectual disability who have a reduced ability to verbalize self-reported pain. They used pain indicators such as problems with behavior, mobility, agitation, aggression, and body language as facial expressions to interpret the level of pain. This finding is supported by (Barney et al., 2020; Lukas et al., 2013; McNeil et al., 2018) that pain assessment in care-receivers with people with intellectual disability is a complex task and can become extremely challenging, especially for those people with diagnosed at the severe and profound levels, as their ability to verbally communicate their pain experience is seriously compromised. According to the study of Barney et al. (2020), it is recommended to use assessment tools like self-report scales such as pain scales with pictures such faces or pyramid scales. These pain scales may have limited validity in use towards people with intellectual disability, but nevertheless it is important to try self-reporting towards people with intellectual disability assessed together with other sources of pain information. Studies demonstrate that there are also several tools available as not self-reporting facial pain scales. These pain scales can be used with people with profound intellectual disability such as r-FLACC, Abby Pain Scale, DisDAT among others (Barney et al., 2020; Doody & Bailey, 2017; Weir & Koritsas, 2022).

Communication difficulties are among the underlying causes of challenging behavior in people with intellectual disability (Bowring et al., 2017). It has been shown that the severity of challenging behavior is usually proportional to the extent of communication difficulties experienced by people with intellectual disability (Smith et al., 2020).

### Intellectual disability nurses play a key role in interprofessional collaboration with healthcare services

The informants gave several examples of intellectual disability nurses being advocates for persons with intellectual disabilities when they need medication treatment in emergency rooms and hospitals. An important finding in our study was that intellectual disability nurses encountered numerous cases of people with intellectual disability who received incorrect medication in the emergency room, which could have had serious health consequences. This is supported by a report from the Norwegian Board of Health Supervision (2017) that there are still medication-related errors among people with intellectual disability. According to Van Den Bemt et al. (2007), administering medication to individuals with intellectual disability is prone to serious errors, because the individuals themselves are not alert and therefore cannot respond appropriately when an error occurs. The intellectual disability nurses have a special commitment and readiness to act as advocates for people with intellectual disability. Hence, the immense responsibility of the intellectual disability nurses in their role as mediators between the people with intellectual disability and the physician and the healthcare team in their effort to prevent medication errors. This highlights the importance of the intellectual disability nurse's key role in various situations in collaboration with other health personnel. According to a review study by Jaques et al. (2018), the intellectual disability nurse's competence has a special focus on good communication and interaction with the residents. In addition, they have knowledge of the person's intellectual disability, which is vital in the interprofessional collaboration during medication treatment. The intellectual disability nurse's unique insight into the cognitive and behavioral impairments of the person with intellectual disability contributes to their ability of taking care of the resident's holistic needs (Grong, 2019).

### The need for a better system of medication management

This study uncovered several challenges in promoting safe medication management in residential care homes. This included, among other things, a lack of facilities such as suitable medication rooms, absence of a system for routine medication administration, and in addition a lack of understanding among colleagues with non-health education. Seemingly there is an absence of job differentiation based on competence in residential care homes, which means that the intellectual disability nurses must do many of the same tasks as unskilled colleagues. This has led to increased stress led on by fear of making mistakes. Erickson and Yang’s (2019) study also came to this conclusion. Intellectual disability nurses forced to perform ad-hoc tasks ultimately and detrimentally affected the administration of medication to persons with intellectual disability. The lack of guidelines and frameworks is concerning as this is a complex area of practice, which is also highlighted in international studies in a systematic review where 64 studies were included by Sheerin et al. (2021). For intellectual disability nurses to be able to perform quality-assured medication handling, management must take responsibility for creating justifiable routines for medication management, ensuring to stay in line with the regulations (Norwegian Directorate of Health, 2015). It is not enough to be competent as a health professional when the working conditions provide such great insecurity.

Another important finding revealed poor routines for reporting incidents when various types of mistakes and incidents occurred during the handling of medications. There was no method of officially reporting incidents of deviations within the residential care homes. There were ambiguities among employees regarding who should report incidents. Intellectual disability nurses found this frustrating. When incidents were reported, they were not taken seriously and followed up by the management. Several other studies also show inadequate medication management in primary health care services (Galek et al., 2018; Storli et al., 2016; Wannebo and Sagmo 2013). In line with the Norwegian Board of Health Supervision (2017), which had a nationwide inspection in 2016, major weaknesses were identified in the handling of medications in residential care homes for persons with intellectual disability. It was concluded that reported factors can contribute to serious failures, such as weaknesses in documentation, reporting routines and lack of responsibility for medication management (Norwegian Board of Health Supervision, 2017). The findings of our study indicate that intellectual disability nurses take responsibility in ensuring safety in medication management.

WHO has further developed an action plan in the period 2021-2030. The plan involves three areas of effort to prevent medication damage. This includes vulnerable groups of persons with intellectual disability, polypharmacy, and transitions where the person changes between locations, service levels and health personnel (WHO, 2021). It is important to understand what challenges intellectual disability nurses face throughout the medication management process and how these can be solved.

### Methodological implications

This study included 18 participants who shared their personal experiences about their challenges with medication management for people with intellectual disability in residential care homes. The sample is small and therefore the transferability is limited. But the purpose of a qualitative study is not to generalize, but to obtain a more in-depth understanding of a phenomenon. Credibility of the study can also be strengthened by the informants during the focus group interview, and they also have the opportunity to provide feedback on moderator’s summaries after each question.

## Conclusions

The study shows that intellectual disability nurses encounter numerous challenges in medication management for intellectual disabilities in residential care homes. They are given a great responsibility in the handling of medicines to ensure resident’s safety and are expected to take on the personal responsibility of training unskilled health colleagues in medication management. Furthermore, intellectual disability nurses have a key role in interdisciplinary collaboration to ensure that persons with intellectual disabilities receive the correct medication. Management of residential care homes must facilitate quality work systems, routines and training, thus ensuring professionally sound medication management. There is a shortage of intellectual disability nurses, and the primary health service in Norway must work to ensure that more intellectual disability nurses are employed in the residential care homes. Management must take responsibility for implementing a system that provides sound medication management at all levels to promote patient safety. This study identified critical issues that should be addressed in future research.
